# Risk Factors for Acute Kidney Injury after Congenital Cardiac Surgery in Infants and Children: A Retrospective Observational Study

**DOI:** 10.1371/journal.pone.0166328

**Published:** 2016-11-10

**Authors:** Sun-Kyung Park, Min Hur, Eunhee Kim, Won Ho Kim, Jung Bo Park, Youngwon Kim, Ji-Hyuk Yang, Tae-Gook Jun, Chung Su Kim

**Affiliations:** 1 Department of Anesthesiology and Pain Medicine, Seoul National University Hospital, Seoul, Republic of Korea; 2 Department of Anesthesiology and Pain Medicine, Samsung Medical Center, Sungkyunkwan University School of Medicine, Seoul, Republic of Korea; 3 Department of Thoracic and Cardiovascular Surgery, Samsung Medical Center, Sungkyunkwan University School of Medicine, Seoul, Republic of Korea; Bambino Gesù Children's Hospital, ITALY

## Abstract

Acute kidney injury (AKI) after pediatric cardiac surgery is associated with high morbidity and mortality. Modifiable risk factors for postoperative AKI including perioperative anesthesia-related parameters were assessed. The authors conducted a single-center, retrospective cohort study of 220 patients (aged 10 days to 19 years) who underwent congenital cardiac surgery between January and December 2012. The incidence of AKI within 7 days postoperatively was determined using the Kidney Disease: Improving Global Outcomes (KDIGO) criteria. Ninety-two patients (41.8%) developed AKI and 18 (8.2%) required renal replacement therapy within the first postoperative week. Among patients who developed AKI, 57 patients (25.9%) were KDIGO stage 1, 27 patients (12.3%) were KDIGO stage 2, and eight patients (3.6%) were KDIGO stage 3. RACHS-1 (Risk-Adjusted classification for Congenital Heart Surgery) category, perioperative transfusion and fluid administration as well as fluid overload were compared between patients with and without AKI. Multivariable logistic regression analyses determined the risk factors for AKI. AKI was associated with longer hospital stay or ICU stay, and frequent sternal wound infections. Younger age (<12 months) [odds ratio (OR), 4.01; 95% confidence interval (CI), 1.77–9.06], longer cardiopulmonary bypass (CPB) time (OR, 2.45; 95% CI, 1.24–4.84), and low preoperative hemoglobin (OR, 2.40; 95% CI, 1.07–5.40) were independent risk factors for AKI. Fluid overload was not a significant predictor for AKI. When a variable of hemoglobin concentration increase (>3 g/dl) from preoperative level on POD1 was entered into the multivariable analysis, it was independently associated with postoperative AKI (OR, 6.51; 95% CI, 2.23–19.03 compared with no increase). This association was significant after adjustment with patient demographics, medication history and RACHS-1 category (hemoglobin increase >3g/dl vs. no increase: adjusted OR, 6.94; 95% CI, 2.33–20.69), regardless of different age groups and cyanotic or non-cyanotic heart disease. Prospective trials are required to evaluate whether correction of preoperative anemia and prevention of hemoconcentration may ameliorate postoperative AKI in patients who underwent congenital cardiac surgery.

## Introduction

Infants or children undergoing open cardiac surgery are at risk of developing cardiac surgery-associated acute kidney injury (CS-AKI) [1–3]. Postoperative acute kidney injury (AKI) in pediatric patients is a serious complication associated with adverse outcomes including prolonged mechanical ventilation, prolonged hospital stay, and high morbidity and mortality [1–7]. Previous studies have reported that younger age [1, 3, 4, 7], prematurity [4, 6], longer cardiopulmonary bypass (CPB) time [2–4, 6, 7], a high Risk Adjusted Classification of Congenital Heart Surgery (RACHS-1) score [1, 7], longer vasopressor use [3] and selective cerebral perfusion [8, 9] are associated with CS-AKI in infants and children. Recently, several plasma and urine biomarkers reflecting renal injury have been investigated to facilitate early diagnosis [10–12]. Although these biomarkers are increasingly available at manageable cost in laboratories and the accuracies of such biomarkers are improving, it is still necessary to identify risk factors of CS-AKI that may be clinically modifiable.

There have been several studies of agents including aminophylline and fenoldopam, which have shown promising results in certain subpopulations [13, 14]. Nonetheless, evidence is still lacking and there is no established prevention or effective treatment of AKI after pediatric cardiac surgery [15, 16]. Therefore, a reasonable strategy would be to identify modifiable risk factors in this setting and establish potential interventions which may reduce the risk of AKI. Modifiable risk factors may thus serve as therapeutic goals to prevent AKI in these patients. However, previously identified risk factors in pediatric cardiac patients are not modifiable. Perioperative laboratory and anesthesia-related variables including preoperative anemia [17], hypoalbuminemia [18], and perioperative erythrocyte transfusion [19] that have been proven to be associated with AKI in adult patients, could potentially be considered modifiable factors. In particular, the association between any change in hemoglobin concentrations and postoperative AKI has not been investigated in the pediatric population. Furthermore, the optimal hemoglobin threshold for perioperative red blood cell transfusion in patients with congenital heart disease has not been determined.

Therefore, the objective of this retrospective observational study was to determine potential modifiable risk factors among transfusion and laboratory variables that may identify patients who are at high-risk for AKI following pediatric cardiac surgery. The identification of such risk factors could guide future prospective trials testing whether modification of these risk factors can mitigate the burden of postoperative AKI.

## Materials and Methods

This retrospective observational study was approved by the Samsung Medical Center Institutional Review Board Approval (SMC 2013-12-101-001). The authors retrospectively reviewed the electronic medical charts of 226 consecutive pediatric patients under 18 years of age who underwent elective congenital cardiac or aortic surgery with or without CPB at the Samsung Medical Center between January and December 2012. Our retrospective observational study protocol is registered at http://www.clinicaltrials.gov (NCT02081235) and is compliant to STROBE checklist (S1 Checklist). Procedures for simple and complex cardiac anomalies were included. The need for informed consent was waived for this study, considering its retrospective nature. Patients were excluded if they had missing preoperative serum creatinine (sCr) values (n = 3), preoperative renal replacement therapy (RRT, n = 2), or if they died within 24 hours postoperatively (n = 1). Of the remaining 220 patients, 92 patients (41.8%) developed AKI, as defined by the Kidney Disease: Improving Global Outcomes (KDIGO) criteria [20, 21].

Relevant demographic and perioperative parameters previously shown to be correlated with postoperative AKI were evaluated in this study after literature review [1–4, 6, 7, 22]. The relevant parameters included type of surgery, RACHS-1 category, preoperative angiography, nephrotoxic medication history, perioperative anesthesia-related variables, and laboratory values. Total transfusion amounts per body weight (kg) during surgery and postoperative two days were examined and categorized by 20 ml/kg for packed red blood cells (pRBC) and by 30 ml/kg for fresh frozen plasma (FFP). The calculation of total pRBC transfusion amount included pRBC volume used to prime the CPB circuit. The CPB-priming pRBC was returned in part to the patient during the CPB weaning process according to the patient’s intravascular volume status. Preoperative, postoperative day (POD) 1 and POD2, hemoglobin, albumin and C-reactive protein (CRP) levels were obtained. To evaluate any effect of hemodilution or hemoconcentration, differences between hemoglobin concentrations on POD1 and those on preoperative concentrations were calculated. The amounts of fluid intake during surgery and up to postoperative three days were obtained. The fluid overload index, determined by the following equation [23, 24], was compared between those with and without AKI. The total input was calculated by the sum of total fluid administration (ml) and blood transfusion (ml), while total output was calculated by the sum of urine output, JP and chest tube drainage, and hemodialysis dehydration.

$$Fluid\;overload\;index=\frac{Total\;fluid\;input\;(L)-Total\;fluid\;output\;(L)}{Basic\;Weight\;(kg)}\times 100\;(\%)$$

The development of AKI within the first 7 PODs was the primary outcome variable. Again, AKI was defined according to the KDIGO criteria, which classifies AKI by severity based on the maximal change in sCr from preoperative baseline levels, the criteria of which has also been validated in pediatric patients [20, 25]. All patients who met the KDIGO criteria for stage 1, 2 or 3 were identified as having AKI. The date at which the diagnosis of AKI according to KDIGO criteria was made was also examined to discriminate early versus late AKI. Postoperative RRT was defined as a new need for dialysis after surgery. Postoperative outcome variables included the need for postoperative RRT, length of hospital and ICU stay as well as postoperative complications including incidences of bleeding, wound infection, pneumonia, seizure, and nitric oxide use for pulmonary hypertension.

All operations were performed by one of two surgeons (JY and TJ) experienced in pediatric cardiac surgery for over ten years with more than 200 surgeries per year. Midazolam (0.1–0.3 mg/kg) or thiopental sodium (4–5 mg/kg) was used for induction of anesthesia. Anesthesia was maintained by inhalation anesthetics or total intravenous anesthesia. For CPB, arterial cannulation was performed in the ascending aorta and venous cannulations were bicaval or in the right appendage according to the type of surgery. Non-pulsatile hypothermic CPB was instituted at 2.2 to 2.6 L/min/m^2^. We did not use aprotinin or tranexamic acid for coagulation support. Modified ultrafiltration was conducted in the operating room during CPB weaning process.

### Statistical Analysis

Data were analyzed using the SPSS software version 21.0 (IBM Corp., Armonk, NY, USA). P<0.05 was considered statistically significant for all analyses. A sample size of 200 patients or more was determined to be necessary under the assumption that the expected odds ratio (OR) of AKI development in patients with large intraoperative transfusion amount would be 4.0, with a power of 0.8, and a type I error of 0.05 [26]. For accurate estimation, the sample size was also validated according to a target number of outcome events of ten per independent predictor [27]. For the current study, it was estimated 200 patients or more are necessary to permit unbiased accommodation of five or fewer predictive variables in a multiple logistic regression model (estimated 25% incidence of postoperative AKI) [27]. Categorical variables were reported as absolute number (*n*) and relative frequency (%), and continuous variables were reported as the mean (standard deviation) or median (interquartile range). Fisher’s exact test or χ^2^ test was used to compare the incidence variables according to their expected counts. Comparisons of continuous variables were performed with Mann-Whitney *U* tests or unpaired *t*-tests according to their distribution. Logistic regression models were used to identify univariable and multivariable predictors for AKI. After univariable logistic regression analysis identified possible risk factors for AKI, the multivariable model only included variables that were found to be significant AKI risk factors upon univariable analysis (*p*<0.05). ORs and 95% confidence intervals (CIs) were generated for each variable to determine the strength of its influence in predicting postoperative AKI.

Three risk prediction models were developed. The first model (model-1) included all significant variables in univariable logistic regression analysis. The second model (model-2) included a multivariable logistic regression analysis of all significant variables in univariable analysis except hemoglobin concentration increase on POD1 from preoperative levels. FFP transfusion amount was not included, as pRBC transfusion amount is a surrogate for overall transfusion amount. The third model (model-3) included all the significant variables in the univariable analysis including hemoglobin increase on POD1 from preoperative levels. Both models considered forward and backward stepwise variable selection. Established cut-offs were used to categorize the continuous variables in the logistic regression models based on results from previous studies [2, 22], or set at the point of maximal sum of sensitivity and specificity.

To further evaluate the association between the intraoperative transfusion amount and postoperative AKI, the adjusted OR of intraoperative pRBC transfusion amount, FFP transfusion amount, and hemoglobin increase from preoperaive value on POD1 were obtained. ORs were adjusted for age, weight, height or body length, gender, complex anomaly, preoperative pulmonary hypertension, hepatomegaly, digoxin administration, diuretics administration, and RACHS-1 category. The effect of hemoglobin increase on POD1 from preoperative value on postoperative AKI was examined on prespecified subgroups, including cyanotic versus non-cyanotic heart disease, different age groups, preoperative hemoglobin concentrations, and POD2 CRP levels.

Missing data were present in less than 2% of records. Missing values for continuous variables were assigned gender-specific median values, and categorical values were assigned the most frequent gender-specific values. To measure and compare the predictive accuracy of the developed risk models, we generated receiver operating characteristic (ROC) curves and compared their C-statistics [28]. Calibration of the risk score was assessed using Hosmer-Lemeshow goodness-of-fit statistics.

## Results

Among the patients who underwent elective pediatric cardiac surgery in 2012 (n = 226), a total of 220 patients were analyzed after exclusion of six patients. Of these 220 patients, 92 (41.8%) developed AKI as defined by the KDIGO criteria, and 18 (8.2%) required RRT within the first postoperative week. Eighty-two patients (52.2%) among 157 neonates or infants developed AKI and 10 patients (15.9%) among 63 children (>12 months) developed AKI. Among the 92 patients who developed AKI, 57 patients (25.9%) were KDIGO stage 1, 27 patients (12.3%) were KDIGO stage 2, and eight patients (3.6%) were KDIGO stage 3. The majority of patients developed AKI on postoperative day one (n = 77, 83.7% among those with AKI) and two (n = 12, 13.0% among those with AKI)(S1 Table)

Patient characteristics and perioperative parameters according to the diagnosis of AKI in our study sample are presented in Table 1 and S2 Table. There were differences in age, weight, height, incidence of preoperative pulmonary hypertension, preoperative digoxin administration, operation time, CPB time, intraoperative blood loss, transfusion amount, preoperative and POD1 hemoglobin, preoperative serum albumin levels, POD2 CRP levels, and the increase in hemoglobin concentration on POD1 from preoperative values (Table 1). The incidences of furosemide and antibiotics use were not different, and other nephrotoxic medications including angiotensin converting enzyme inhibitors were not used.

**Table 1:** Comparison of patient demographics and perioperative clinical parameters between patients with and without postoperative acute kidney injury.

Variable	No AKI (n = 128)	AKI (n = 92)	p-value
**Age (months)**	8 [2–32]	3 [1–6]	<0.001
**Neonate (< 1 month)**	31 (24.2)	29 (31.5)	
**Infant (1 month to 12 months)**	44 (34.4)	53 (57.6)	
**Children (> 12 months)**	53 (41.4)	10 (10.9)	
**Weight (kg)**	8.3 [4.6–12.8]	5.4 [4.2–7.3]	<0.001
**Height or body length (cm)**	71 [57–93]	60 [55–68]	<0.001
**Gender (female; n)**	64 (50.0)	35 (38.0)	0.079
**Preoperative pulmonary hypertension (n)**	11 (8.7)	19 (20.7)	0.011
**Tricuspid regurgitation more than mild degree**	61 (49.2)	34 (37.0)	0.073
**Hepatomegaly (n)**	7 (5.5)	9 (9.8)	0.294
**Preoperative digoxin administration (n)**	2 (1.6)	7 (7.6)	0.037
**Preoperative furosemide administration (n)**	28 (21.9)	27 (29.3)	0.207
**Preoperative antibiotics administration (n)**			
**Cephalsporin**	53 (41.4)	36 (39.1)	0.734
**Tazocin**	9 (7.0)	6 (6.5)	0.882
**Preoperative cardiac CT scan (n)**	33 (25.8)	31 (33.7)	0.202
**Preoperative cardiac angiography**	28 (21.9)	19 (20.7)	0.827
**Radiocontrast dose used in cardiac angiography (ml/kg)**	4 [3–5]	3 [2–5]	0.362
**RACHS-1 risk category 1–2 / 3–4 / 5–6 (n)**	84 (65.6) / 44 (34.4) / -	62 (67.4) / 30 (32.6) / -	0.108
**Resternotomy (n)**	37 (28.9)	23 (25.0)	0.521
**Anesthesia-related factors**			
**Operation time (min)**	252 [210–318]	271 [233–350]	0.035
**CPB time (min)**	85 [58–116]	94 [68–147]	0.022
**Intraoperative blood loss (ml/kg)**	18.0 [10.2–35.2]	21.6 [15.2–41.7]	0.018
**Estimated blood volume (ml)**	664 [374–1026]	440 [351–572]	<0.001
**Intraoperative excessive blood loss (n)**	26 (20.5)	23 (25.0)	0.427
**Intraoperative urine output (ml/kg)**	15.0 [9.8–23.4]	13.1 [6.0–22.9]	0.207
**Intraoperative fluid intake (ml/kg/hr)**	16.8 [14.9–29.5]	16.9 [15.0–28.6]	0.204
**Intraoperative transfusion amount (ml/kg)**			
**Packed red blood cells**	41.9 [26.6–67.4]	62.6 [47.4–87.8]	<0.001
**Fresh frozen plasma**	2.7 [0–32.1]	42.1 [0–56.1]	<0.001
**Platelet concentrate**	0 [0–6.6]	0 [0–7.8]	0.279
**Cryoprecipitate**	0 [0–0]	0 [0–0]	0.475
**Laboratory variables**			
**Hemoglobin (g/dl)**			
**Preoperative**	12.9 [11.6–14.0]	11.9 [10.7–13.0]	<0.001
**POD1**	11.9 [10.8–13.1]	13.2 [11.8–14.7]	<0.001
**POD2**	12.2 [11.3–13.4]	12.6 [11.5–13.6]	0.142
**Hemoglobin increase from preoperative level on POD1**			<0.001
**No increase or decrease**	89 (69.5)	32 (34.8)	
**0 < increase < 3 (g/dl)**	33 (25.8)	38 (41.3)	
**3 (g/dl) < increase**	6 (4.7)	22 (23.9)	
**Serum albumin (g/dl)**			
**Preoperative**	4.4 [4.1–4.6]	4.1 [3.8–4.4]	<0.001
**POD1**	4.3 [3.9–4.5]	4.3 [4.0–4.6]	0.507
**POD2**	4.1 [3.6–4.4]	4.0 [3.6–4.3]	0.405
**Total fluid intake during postoperative three days (ml/kg/day)**	262 [229–266]	256 [244–266]	0.690
**Fluid overload index during postoperative three days (%)**	4.17 (-5.07–7.23)	-5.59 (-9.73–4.41)	<0.001
**Fluid overload index > 7.2% during postoperative three days (n)**	32 (25.0%)	14 (15.2%)	0.078
**C-reactive protein > 10 mg/dl (n)**			
**POD2**	8 (6.3)	9 (9.8)	0.333

The values are presented as the median [interquartile range], or the number of patients (%) per group.

AKI = acute kidney injury, RACHS = Risk-Adjusted classification for Congenital Heart Surgery, CPB = cardiopulmonary bypass, POD = postoperative day.

The clinical outcomes were compared between patients with and without postoperative AKI in Table 2. Patients with AKI had significantly longer length of hospital stay, postoperative ICU stay and significantly higher incidence of sternal wound infection. The one-year mortality was significantly higher in patients with AKI than in those without AKI (Table 2).

**Table 2:** Comparison of clinical outcomes between patients with and without postoperative acute kidney injury.

Variable	No AKI (n = 128)	AKI (n = 92)	p-value
**Length of hospital stay (days)**	13 [9–20]	14 [11–25]	0.007
**Postoperative ICU stay (days)**	4 [2–9]	7 [5–11]	<0.001
**Postoperative mechanical ventilation (days)**	2.2 [1.5–5.0]	4.8 [3.2–8.5]	<0.001
**Postoperative renal replacement therapy (n)**	1 (0.8)	4 (4.3)	0.164
**Postoperative bleeding event (n)**	-	3 (3.3)	0.070
**Sternal wound infection (n)**	-	5 (5.5)	0.012
**Postoperative pneumonia (n)**	5 (3.9)	8 (8.8)	0.154
**Postoperative nitric oxide use for pulmonary hypertension (n)**	16 (12.5)	20 (22.0)	0.062
**Postoperative seizure (n)**	1 (0.8)	3 (3.3)	0.310
**In–hospital mortality (n)**	0	1 (1.1%)	0.418
**One-year mortality (n)**	0	4 (4.3%)	0.029

The values are presented as the median [interquartile range], or the number of patients (%) per group.

AKI = acute kidney injury.

**Fig 1:**
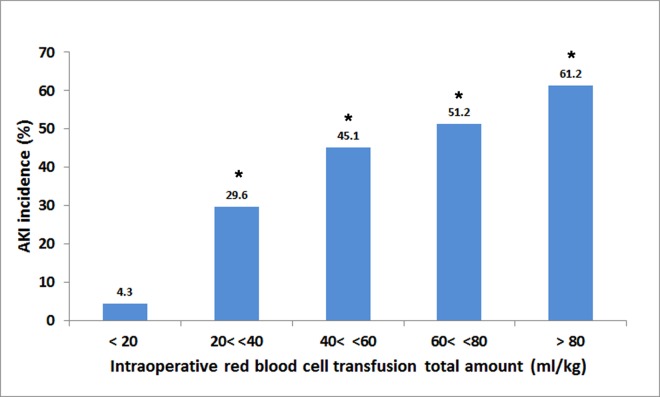
Incidences of postoperative acute kidney injury according to categorized total amount (mg/kg) of intraoperative red blood cell transfusion. *Significantly different from the patients with intraoperative transfusion amount <20 ml/kg.

The results of both univariable and multivariable analyses of risk factors for AKI within all KDIGO stages are displayed in Table 3 and S3 Table. Intraoperative red blood cell transfusion amounts (ml/kg) were associated with graded increase in the risk of AKI development (>80 ml/kg, OR, 34.74; 95% CI, 4.32–279.38 compared with <20 ml/kg)(Fig 1)(S3 Table). Two different multivariable logistic analyses were performed with or without the parameter of hemoglobin increase from preoperative level on POD1 (Table 3). Among the potential risk factors determined by univariable analysis (Risk model-1), the independent risk factors for AKI included age <12 months, CPB time >120 min, preoperative hemoglobin <11.0 g/dl, and POD2 CRP >5.0 mg/dl when the parameter of hemoglobin increase was not included (Risk model-2). When the parameter of hemoglobin increase was included, age <12 months, CPB time >120 min, and hemoglobin increase were significant (Risk model-3). The results for both forward and backward stepwise variable selection were identical.

**Table 3:** Multivariable analysis of predictors for postoperative acute kidney injury.

Covariate	β-coefficient	Odds Ratio	95% CI	p-value	Risk Score
**Risk model-2**					
**Age < 12 months**	1.44	4.24	1.82–9.89	0.001	4
**CPB time > 120 min**	0.74	2.09	0.96–4.56	0.063	2
**Preoperative hemoglobin < 11.0 g/dl**	0.89	2.43	1.08–5.46	0.032	2
**Constant**	-2.08			<0.001	
**Risk model-3**					
**Age < 12 months**	1.20	3.33	1.39–7.96	0.007	3
**CPB time > 120 min**	0.97	2.64	1.18–5.91	0.018	3
**Hemoglobin increase from preoperative level on POD1 (g/dl)**					
**No increase or decrease**			Baseline		
**0< increase < 3 (g/dl)**	1.06	2.87	1.40–5.89	0.004	3
**3 (g/dl) < increase**	1.88	6.56	2.21–19.52	0.001	7
**Constant**	-2.38			<0.001	

CI = confidence interval, CPB = cardiopulmonary bypass, POD = postoperative day.

**Fig 2:**
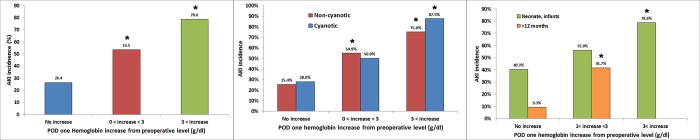
Incidences of postoperative acute kidney injury. The incidences were shown according to categorized hemoglobin increase on postoperative day one from preoperative level (g/dl) in all patients (left), in patients with non-cyanotic and cyanotic heart disease (middle), and in patient subgroup of neonates or infants and children > 12 months (right).

The hemoglobin increase from preoperative values on POD1 was significant after adjustment for patient demographics, baseline medical conditions, and RACHS-1 category, while intraoperative pRBC and FFP transfusion amounts were not (Table 4). A hemoglobin increase from preoperative value on POD1 >3 g/dl was associated with increased AKI in all subgroups listed in methods except the subgroup with preoperative hemoglobin <11.0 g/dl (Fig 2).

**Table 4:** Association between intraoperative transfusion amount and postoperative acute kidney injury.

Variable	No AKI	AKI	Crude OR (95% CI)	p-value	Adjusted OR^a^ (95% CI)	p-value
**pRBC transfusion amount during surgery and postoperative two days**						
**< 20 ml/kg**	22 (17.2)	1 (1.1)	Reference		Reference	
**20 to 39 ml/kg**	37 (28.9)	16 (17.4)	9.51 (1.18–76.77)	0.034	2.32 (0.24–22.42)	0.466
**40 to 59 ml/kg**	28 (21.9)	23 (25.0)	18.07 (2.26–144.4)	0.006	3.31 (0.32–34.47)	0.316
**60 to 79 ml/kg**	19 (14.8)	22 (23.9)	25.47 (3.13–207.18)	0.002	4.37 (0.39–48.78)	0.231
**> 80 ml/kg**	22 (17.2)	30 (32.6)	30.00 (3.76–239.69)	0.001	4.79 (0.40–54.85)	0.216
**FFP transfusion amount during surgery and postoperative two days**						
**0 to 29 ml/kg**	92 (71.9)	33 (35.3)	Reference		Reference	
**30 to 59 ml/kg**	21 (16.4)	39 (42.4)	5.18 (2.67–10.05)	<0.001	2.46 (1.03–5.88)	0.044
**> 60 ml/kg**	15 (11.7)	20 (21.7)	3.72 (1.71–8.10)	0.001	0.95 (0.27–3.36)	0.932
**Hemoglobin increase from preoperative value on POD1 (g/dl)**						
**No increase or decrease**	89 (69.5)	32 (34.8)	Reference		Reference	
**0< increase < 3 (g/dl)**	33 (25.8)	38 (41.3)	3.20 (1.73–5.94)	<0.001	2.36 (1.17–4.77)	0.017
**3 (g/dl) <increase**	6 (4.7)	22 (23.9)	10.20 (3.79–27.42)	<0.001	6.94 (2.33–20.69)	0.001

The values are presented as the number of patients (%) per group.

AKI = acute kidney injury, OR = odds ratio, CI = confidence interval, pRBC = packed red blood cells, FFP = fresh frozen plasma, POD = postoperative day.

^a^Odds ratio are adjusted for age, weight, height or body length, gender, complex anomaly, preoperative pulmonary hypertension, hepatomegaly, digoxin administration, diuretics administration, RACHS-1 category.

We developed risk-prediction models for postoperative AKI with the results of the univariable (S3 Table, Risk model-1) and multivariable analyses (Table 3, Risk model-2, 3). Our three risk models produced good calibration and discrimination in terms of c-statistics in our study sample (area under the receiver operating characteristic curve of model-3: 0.77, 95% CI, 0.71 to 0.84) (S4 Table).

## Discussion

The aim of our study was to find potentially modifiable risk factors among transfusion and laboratory variables for postoperative AKI in patients undergoing pediatric cardiac surgery. In addition to previously known risk factors including young age and long CPB time, preoperative anemia, elevated CRP levels on POD2, and an increase in hemoglobin concentrations from preoperative values were identified as new independent predictors of postoperative AKI in these patients. An increase in hemoglobin concentration compared to preoperative values was a significant predictor for AKI after adjusting for patient demographics, baseline medical status and RACHS-1 category. In a subgroup analysis, an increase in hemoglobin concentrations was significant only in patients with preoperative hemoglobin concentration ≥11.0 g/dl and not in those with <11.0 g/dl. These results suggest that hemoconcentration may be associated with AKI after pediatric cardiac surgery.

The incidence of CS-AKI in our pediatric population with a mixed sample of neonates, infants, and children was 41.8% as defined by the KDIGO criteria, which was similar to previous studies ranging from 15% to 64% [1–6]. The incidence of pediatric CS-AKI varies based on the different definitions used and the age of the study population [1–6]. The KDIGO criteria was chosen to be the primary outcome variable in the present study, but other criteria including the pRIFLE (pediatric Risk, Injury, Failure, Loss of function and End-stage renal disease) criteria and the AKIN (acute kidney injury network) criteria could be used to diagnose AKI in pediatric patients. A previous study that compared these three criteria in pediatric cardiac patients reported that the pRIFLE system was the most sensitive test in detecting AKI and the AKIN system was the most specific and detected mostly high-risk patients [25]. The study reported that the KDIGO classification system fell between pRIFLE and AKIN in terms of performance. Moreover, the KDIGO criteria were validated in a broad pediatric critical care population including cardiac patients [20]. It is also important to identify that the baseline creatinine in neonates is often a reflection of the maternal creatinine when it was obtained during the first few days of life [29]. This may result in a higher baseline creatinine and thus result in the underestimation of the degree of kidney injury.

The incidence of AKI after pediatric cardiac surgery was reported to be higher in patients with younger age [1, 3, 4, 7], and in premature patients [4, 6]. Multivariable analysis of the present study showed that age of less than 12 months was an independent predictor of AKI after cardiac surgery. This result may be associated with the intrinsic immaturity of the renal tubule and its reduced ability to adapt to post-CPB inflammation and ischemia reperfusion injury of the kidney in younger patients [30, 31]. In particular, children less than 2 years of age may be more prone to inflammatory and ischemic insults, because maximal glomerular filtration rate is achieved only after the age of 2 years [32].

In our study, a longer CPB duration (>120 min) was independently associated with postoperative AKI, which was consistent with previous studies [2–4, 6, 7]. Long CPB time may be associated with more severe ischemia and progressive inflammation, which increases the risk of AKI [15]. The RACHS-1 score has been reported to be associated with postoperative AKI [1, 7], but it was not significant in our study. This may be due to the relative absence of high category surgeries (category-5, 6) in our study sample and different risks according to the RACHS-1 score may not have been revealed in our study.

Although not significant in multivariable analysis, preoperative pulmonary hypertension was associated with pediatric CS-AKI in our study. A previous study reviewing 105 adult patients who were hospitalized with pulmonary arterial hypertension with acute right-sided heart failure showed that AKI occurred in 32% of patients and was strongly associated with 30-day mortality [33]. Venous congestion is thought to be a hypothetical mechanism leading to AKI in patients with pulmonary hypertension. Previous studies have demonstrated that venous congestion, evident from the increased right atrial size and central venous pressure, is an important hemodynamic factor driving AKI [33, 34].

Postoperative CRP as a continuous variable was found to be associated with postoperative AKI on univariate analysis. However, its categorized variable with cutoff of normal reference (> 10 mg/dl) was not significant. Previous studies reported perioperative CRP was a significant predictor for AKI and clinical outcomes in adult cardiac surgery or coronary intervention [18, 35, 36]. CRP is a nonspecific marker of systemic inflammation produced within 6 hours of surgical stress and peaks at 36 to 60 hours after surgery. The levels of CRP are known to be associated with the severity of systemic inflammatory response [37].

Preoperative anemia was revealed to be an independent predictor of postoperative AKI. This is important because anemia is a potentially modifiable risk factor [17]. Many of the younger infants may perhaps be at their physiological nadir of hematocrit, which may in part explain the etiology of preoperative anemia [38]. These patients are often getting elective surgery with laboratory evaluation only a few days before surgery. Prospective studies are required to evaluate whether preoperative correction of anemia may mitigate postoperative AKI in neonates or premature infants. Preoperative administration of recombinant human erythropoietin may correct preoperative anemia and, thus, the need for transfusion [38, 39], although this practice has not yet been widely adopted.

Another major finding of this study was that there was a graded and significant association between AKI after pediatric cardiac surgery and the increase in hemoglobin concentrations on POD1 compared to preoperative values. The increase in hemoglobin was an independent predictor of multivariable analysis and was significant after vigorous adjustment for patient demographics and baseline medical conditions, while pRBC and FFP transfusion amounts were not significant after adjustment. In the subgroup analysis, the increase in hemoglobin was significant in patients with preoperative hemoglobin ≥ 11.0 g/dl. As the amounts of fluid intake during operation and postoperative three days and the incidences of fluid overload were similar between those with and without AKI, these results suggest that hemoconcentration developed from the large number of pRBC transfusions during pediatric cardiac surgery and may contribute to AKI. However, postoperative hematocrit values also vary based on the procedure performed. A preoperative cyanotic patient may have an elevated hematocrit, but then have either still elevated or normal hematocrit after surgical repair. Therefore, postoperative hematocrit values are also associated with the postoperative physiology according to the surgical procedure performed. From this perspective, the surgical procedure could be an effect modifier for AKI. Further studies with sufficient number of patients and surgical type may answer this question.

A previous study has reported that both hemoconcentration and hemodilution during CPB were associated with adult CS-AKI [40]. We examined both the increase and decrease in hemoglobin concentration as potential predictors for CS-AKI, but hemoglobin decrease was not significant. It is not certain why only the association between hemoconcentration and postoperative AKI is revealed in our study of pediatric cardiac patients. It may be because hemodilution occurred in fewer patients in our study sample than hemoconcentration did.

There are possible pathophysiologic explanations as to why hemoconcentration may provoke postoperative AKI in infants and children. First, elevated hematocrit increases the blood viscosity and may impair renal microvascular circulation due to decreased blood flow rate [41, 42]. There is an exponential relationship between the hematocrit value and blood viscosity and blood viscosity becomes increasingly sensitive to hematocrit alteration at higher levels of hematocrit [42]. The blood viscosity may increase further in patients with cyanotic heart disease whose baseline hematocrit is already elevated. In addition, oxygen-free radicals affect RBC aggregability [43], which may result in impaired tissue perfusion. The hypoperfusion due to decreased microvascular flow may contribute to the renal ischemic damage especially during cardiac surgery where low cardiac output and cardiogenic shock may frequently occur [44]. Second, these ischemic insults due to decreased renal perfusion may elicit subsequent ischemia-reperfusion injury, inflammation and oxidative stress by free radical toxicity [45], all of which may contribute to the development of AKI.

Sensitivity analyses were performed in different subgroups to evaluate the role of potential confounders. This association was significant between both those with cyanotic and non-cyanotic cardiac patients in the present study. It was also consistent across different age groups. However, this association was significant only when preoperative hemoglobin was greater than 11.0 g/dl, also suggesting that these associations are related to hemoconcentration. Prospective trials are required to confirm these associations and the proper transfusion target of hemoglobin need to be established.

### Limitations

The authors acknowledge that the present study is not without limitations. First, the retrospective study design precludes the suggestion of any causal inference. Bias from unknown or unmeasured confounders may have influenced the results. For example, intraoperative blood loss volume should be obtained from gauze count, discard suction volume (except irrigation fluid volume), chest tube output at the end of surgery and total intraoperative volume of salvaged red blood cells [46]. However, it is difficult to measure all these variables routinely in the OR and using the recorded blood loss volume in the study may carry a significant error. Second, our study enrolled a relatively small number of patients in a single center and thus external validity is limited. The risk score was developed to test the predictability of our risk factors but was not validated externally. Third, several factors affect sCr concentration in neonates, such as a decrease in sCr during the first week of life, bringing into question of the accuracy of AKI diagnosis in neonates [4]. However, diagnostic criteria using sCr change including the KDIGO criteria were validated in pediatric patients including neonates by demonstrating the association between AKI and clinical outcomes [20, 25]. Fourth, AKI was determined according to the KDIGO criteria using increase in sCr of more than 1.5 times the baseline up to seven days in the present study [47]. A further time beyond 48 hours following cardiac surgery makes AKI to be less likely a result of preoperative and operative variables and more likely to be due to secondary factors or insults post-operatively that either lead to development of AKI or progression of AKI that occurred due to pre-op and operative factors. However, in our review of creatinine data to examine the onset day of AKI in individual patients, 96.7% of patients developed AKI during postoperative two days. Fifth, most of the patients who developed postoperative AKI were KDIGO stage 1. Stage 1 AKI often recovers spontaneously without any specific intervention and rarely increases hospital mortality. Thus, the association with one-year mortality after congenital cardiac surgery may be more related to the decreased cardiac function or cardiac surgery-related complications rather than postoperative AKI. Fifth, urine output criteria were not used to diagnose AKI. Accurate urine output data of the 6-hour intervals at the general ward were not available. Although most of the previous studies did not use urine output criteria to diagnose AKI, the incidence of AKI could be changed if it is included.

### Conclusion

Preoperative low hemoglobin concentration was independently associated with AKI after congenital cardiac surgery, in addition to previously known risk factors including longer CPB time and younger age. When an increase in POD1 hemoglobin concentrations compared to preoperative hemoglobin concentrations was included in the analysis, it remained an independent predictor of AKI, suggesting the possible contribution of hemoconcentration in the pathogenesis of AKI after pediatric cardiac surgery. These risk factors may assist in risk evaluation for postoperative AKI. Prospective trials, however, are required to evaluate whether correction of preoperative anemia and prevention of hemoconcentration may ameliorate postoperative AKI in patients who underwent congenital cardiac surgery.

## Supporting Information

S1 Checklist A STROBE checklist for the present study. (DOC)

S1 File A dataset for the present study. (XLSX)

S1 Table The days of onset of postoperative AKI. (DOC)

S2 Table Comparison of detailed surgery types between patients with and without postoperative acute kidney injury. (DOC)

S3 Table Univariate analysis of predictors for acute kidney injury (Risk model-1). (DOC)

S4 Table Receiver operating characteristic curves for prediction of acute kidney injury according to the risk models of the present study. AUC = area under the receiver operating characteristic curves, CI = confidence interval.
(DOC)
