# Supplementary material for: Risk Factors for Acute Kidney Injury after Congenital Cardiac Surgery in Infants and Children: A Retrospective Observational Study
Source: PLoS One. 2016 Nov 10;11(11):e0166328. doi: 10.1371/journal.pone.0166328 (PMC5104485; doi:10.1371/journal.pone.0166328)
Supplement: S1 Table — (DOC) [file pone.0166328.s003.doc]

**Table S1.** The onset day of postoperative AKI.

| One of AKI | Patients who develop AKI | % among patients with AKI |
| --- | --- | --- |
| POD1 | 77 | 83.7% |
| POD2 | 12 | 13.0% |
| POD3 | 1 | 1.1% |
| POD4 | 1 | 1.1% |
| POD5 | 1 | 1.1% |
| POD6 | 0 | - |
| POD7 | 0 | - |

AKI = acute kidney injury, POD = postoperative days.
