# Supplementary material for: Risk Factors for Acute Kidney Injury after Congenital Cardiac Surgery in Infants and Children: A Retrospective Observational Study
Source: PLoS One. 2016 Nov 10;11(11):e0166328. doi: 10.1371/journal.pone.0166328 (PMC5104485; doi:10.1371/journal.pone.0166328)
Supplement: S2 Table — (DOC) [file pone.0166328.s004.doc]

**Table S2. Comparison of detailed surgery types between patients with and without postoperative acute kidney injury**

| Variable | No AKI (n=128) | AKI (n=92) | p-value |
| --- | --- | --- | --- |
| ASD closure, simple and combined | 55 (43.0) | 42 (45.7) | 0.693 |
| VSD closure, simple and combined | 75 (58.6) | 69 (75.0) | 0.012 |
| AVSD closure | 8 (6.3) | 7 (7.6) | 0.788 |
| Total repair of tetralogy of Fallot | 6 (4.7) | 5 (5.4) | 0.999 |
| Coarctation repair | 8 (6.3) | 11 (12.0) | 0.137 |
| Pulmonary outflow tract augmentation | 18 (14.4) | 13 (14.1) | 0.989 |
| Repair of double-outlet right ventricle | 5 (3.9) | 8 (8.7) | 0.137 |
| Repair of total anomalous pulmonary veins | 5 (3.9) | 7 (7.6) | 0.233 |
| Partial anomalous pulmonary venous connection surgery | 4 (3.1) | 1 (1.1) | 0.403 |
| Ross procedure | 0 (0) | 2 (2.2) | 0.174 |
| Right ventricle to pulmonary artery conduit | 11 (8.6) | 9 (9.8) | 0.762 |
| Pulmonary valve replacement | 2 (1.6) | - | 0.511 |
| Pulmonary artery banding | 6 (4.7) | 2 (2.2) | 0.473 |
| Repair of pulmonary artery stenosis | 27 (21.1) | 20 (21.7) | 0.908 |
| Tricuspid valvulotomy or valvuloplasty | 46 (35.9) | 44 (47.8) | 0.077 |
| Fontan procedure | 2 (1.6) | - | 0.511 |
| Rastelli operation | 6 (4.7) | 2 (2.2) | 0.473 |
| Arterial switch operation | 2 (1.6) | 2 (2.2) | 0.999 |
| Tricuspid valve repositioning for Ebstein anomaly | 2 (1.6) | 1 (1.1) | 0.999 |
| Repair of truncus arteriosus | 1 (0.8) | - | 0.999 |
| Bidirectional cavopulmonary shunt | 4 (3.1) | 3 (3.3) | 0.999 |
| Modified Blalock-Taussig shunt | 4 (3.1) | 3 (3.3) | 0.999 |

Data are presented as number (%).

ASD = atrial septal defect, VSD = ventricular septal defect, AVSD = atrio-ventricular septal defect.
