# Supplementary material for: Risk Factors for Acute Kidney Injury after Congenital Cardiac Surgery in Infants and Children: A Retrospective Observational Study
Source: PLoS One. 2016 Nov 10;11(11):e0166328. doi: 10.1371/journal.pone.0166328 (PMC5104485; doi:10.1371/journal.pone.0166328)
Supplement: S3 Table — (DOC) [file pone.0166328.s005.doc]

**Table S3**. Univariate analysis of predictors for acute kidney injury (Risk model-1)

| Variable | No AKI (n=128) | AKI by KDIGO (n=92) | Odds Ratio  (95% CI) | *p*-value | Risk Score | AUC for single variable |
| --- | --- | --- | --- | --- | --- | --- |
| Age < 12 months | 72 (56.3) | 82 (89.1) | 6.38 (3.03 – 13.42) | <0.001 | 6 | 0.66 |
| Weight < 6 kg | 43 (33.6) | 52 (56.5) | 2.57 (1.48 – 4.46) | 0.001 | 3 | 0.62 |
| Height < 60 cm | 36 (28.1) | 46 (50.0) | 2.56 (1.46 – 4.48) | 0.001 | 3 | 0.60 |
| Pulmonary hypertension | 11 (8.7) | 19 (20.7) | 2.75 (1.24 – 6.10) | 0.013 | 3 | 0.56 |
| Preoperative digoxin administration | 2 (1.6) | 7 (7.6) | 5.15 (1.04 – 25.38) | 0.044 | 5 | 0.53 |
| Tricuspid valvuloplasty | 46 (35.9) | 44 (47.8) | 1.63 (0.95 – 2.82) | 0.078 | 2 | 0.56 |
| Operation time > 420 min | 9 (7.0) | 13 (14.1) | 2.18 (0.89 – 5.33) | 0.089 | 2 | 0.53 |
| Surgery with CPB | 118 (92.2) | 91 (98.9) | 7.71 (0.97 – 61.34) | 0.054 | 8 | 0.53 |
| CPB time > 120 min | 26 (22.0) | 37 (40.7) | 2.43 (1.33 – 4.43) | 0.004 | 2 | 0.58 |
| Intraoperative blood loss > 50 ml/kg | 16 (12.6) | 17 (18.5) | 1.57 (0.75 – 3.31) | 0.232 | 2 | 0.52 |
| pRBC transfusion amount during operation and postoperative two days |  |  |  |  |  | 0.68 |
| < 20 ml/kg | 22 (17.2) | 1 (1.1) | Reference |  |  |  |
| 20 to 39 ml/kg | 37 (28.9) | 16 (17.4) | 9.51 (1.18 – 76.77) | 0.034 | 10 |  |
| 40 to 59 ml/kg | 28 (21.9) | 23 (25.0) | 18.07 (2.26 – 144.4) | 0.006 | 18 |  |
| 60 to 79 ml/kg | 19 (14.8) | 22 (23.9) | 25.47 (3.13 – 207.18) | 0.002 | 25 |  |
| > 80 ml/kg | 22 (17.2) | 30 (32.6) | 30.00 (3.76 – 239..69) | 0.001 | 30 |  |
| FFP transfusion amount during operation and postoperative two days |  |  |  |  |  | 0.67 |
| 0 to 29 ml/kg | 92 (71.9) | 33 (35.3) | Reference |  |  |  |
| 30 to 59 ml/kg | 21 (16..4) | 39 (42.4) | 5.18 (2.67 – 10.05) | <0.001 | 5 |  |
| > 60 ml/kg | 15 (11.7) | 20 (21.7) | 3.72 (1.71 – 8.10) | 0.001 | 4 |  |
| Preoperative hemoglobin < 11.0 mg/dl | 14 (10.9) | 27 (29.3) | 3.38 (1.66 – 6.91) | 0.001 | 3 | 0.59 |
| Hemoglobin increase from preoperative value on POD1 (g/dl) |  |  |  |  |  | 0.70 |
| No increase or decrease | 89 (69.5) | 32 (34.8) | Reference |  |  |  |
| 0< increase < 3 (g/dl) | 33 (25.8) | 38 (41.3) | 3.20 (1.73 – 5.94) | <0.001 | 3 |  |
| 3 (g/dl) < increase | 6 (4.7) | 22 (23.9) | 10.20 (3.79 – 27.42) | <0.001 | 10 |  |
| Preoperative albumin < 4.0 g/dl | 22 (17.2) | 34 (37.0) | 2.82 (1.51 – 5.27) | 0.001 | 3 | 0.60 |
| Fluid overload PDO1 | 4 (3.1%) | 7 (7.6%) | 2.55 (0.73 – 8.99) | 0.145 | - | - |
| Fluid overload POD2 | 7 (5.5%) | 8 (8.7%) | 1.65 (0.58 – 4.71) | 0.353 | - | - |
| Fluid overload PDO3 | 4 (3.1%) | 6 (6.5%) | 2.16 (0.59 – 7.89) | 0.243 | - | - |
| Fluid overload during postoperative three days | 32 (25.0%) | 14 (15.2%) | 0.54 (0.27 – 1.08) | 0.081 | - | - |
| C-reactive protein on POD2 > 10 mg/dl | 8 (6.3) | 9 (9.8) | 1.63 (0.60 – 4.39) | 0.337 | - | - |

The values are presented as the number of patients (%) per group.

CI = confidence interval, AUC = area under the ROC curve, CPB = cardiopulmonary bypass, pRBC = packed red blood cells, FFP = fresh frozen plasma, POD = postoperative day.
