# Supplementary material for: Risk Factors for Acute Kidney Injury after Congenital Cardiac Surgery in Infants and Children: A Retrospective Observational Study
Source: PLoS One. 2016 Nov 10;11(11):e0166328. doi: 10.1371/journal.pone.0166328 (PMC5104485; doi:10.1371/journal.pone.0166328)
Supplement: S4 Table — AUC = area under the receiver operating characteristic curves, CI = confidence interval. (DOC) [file pone.0166328.s006.doc]

**Table S4. Comparison of the area under the ROC curve (AUC) of risk models in predicting postoperative acute kidney injury**

|  |  |  | Hosmer-Lemeshow Goodness of fit | | |
| --- | --- | --- | --- | --- | --- |
| Risk score | AUC | 95% CI | χ^2^ statistics | df | p-value |
| Risk model-1 | 0.76 | 0.70 – 0.83 | 1.349 | 8 | 0.995 |
| Risk model-2 | 0.76 | 0.70 – 0.82 | 3.594 | 5 | 0.609 |
| Risk model-3 | 0.77 | 0.71 – 0.84 | 4.999 | 8 | 0.758 |

CI = confidence interval, df= degree of freedom.

Risk model-1 included all significant variables in univariate logistic regression analysis (Supplemental Table 2).

Risk model-2 and 3 were developed from the results of Table 3. Risk model-2 included a multivariate logistic regression analysis of all significant variables in univariate analysis except hemoglobin concentration increase on POD1 from preoperative levels. Risk model-3 included all the significant variables in the univariate analysis including hemoglobin increase on POD1 from preoperative levels.
